# Experimental approaches to study plant cell walls during plant-microbe interactions

**DOI:** 10.3389/fpls.2014.00540

**Published:** 2014-10-14

**Authors:** Ye Xia, Carloalberto Petti, Mark A. Williams, Seth DeBolt

**Affiliations:** ^1^Department of Horticulture, University of KentuckyLexington, KY, USA

**Keywords:** cell imaging, metabolic profiling, phytobiome, plant-microbe interaction, cell wall

## Abstract

Plant cell walls provide physical strength, regulate the passage of bio-molecules, and act as the first barrier of defense against biotic and abiotic stress. In addition to providing structural integrity, plant cell walls serve an important function in connecting cells to their extracellular environment by sensing and transducing signals to activate cellular responses, such as those that occur during pathogen infection. This mini review will summarize current experimental approaches used to study cell wall functions during plant-pathogen interactions. Focus will be paid to cell imaging, spectroscopic analyses, and metabolic profiling techniques.

## Introduction

The plant cell wall is a complex network consisting of diverse polysaccharides, lignin, and proteins (Mutwil et al., 2008). It provides physical strength, maintains cell shape, resists internal turgor pressure, regulates cell differentiation and growth, mediates bio-molecule transit, and serves as the first barrier of defense against biotic and abiotic stress (Knox, 2008; Collinge, 2009; Endler and Persson, 2011). Cell walls are highly dynamic and are capable of modifying their structural and chemical compositions to maintain functionality during developmental growth (Brewin, 2004; Somerville et al., 2004; Gorshkova et al., 2013; Bellincampi et al., 2014). In addition to its structural roles, plant cell walls serve an important function in connecting extracellular and intracellular environments by sensing and transducing signals, and activating cellular responses (Pogorelko et al., 2013) to environmental change and pathogen attack (Aziz et al., 2004; Vorwerk et al., 2004; Hématy et al., 2009). At pathogen infection sites plants generally accumulate callose, phenolic compounds, and lignin (Underwood, 2012), and in some cases metabolites and proteins that can directly inhibit the growth of pathogens (Vorwerk et al., 2004; Haas et al., 2009). The importance of plant cell wall integrity and cell wall-mediated resistance during plant-microbial interaction has been demonstrated, but the related components and signaling pathways have not been fully elucidated (Mellersh and Heath, 2001; Collinge, 2009; Hématy et al., 2009).

This mini review will summarize current experimental approaches that may be used as tools to study the cell wall with a focus on techniques that could be applied during the interaction between a plant and an interacting microbe. In particular, focus will be given to techniques for assessing changes in metabolites during plant-microbe interaction as well as techniques for imaging the cell wall. We are particularly interested in how the phytobiome, including mutualistic endophytes, pathogens and symbionts alike interact with the plants central architectural framework, the cell wall, and how this information could be harnessed for isolation of new herbicides (Xia et al., 2014) and/or plant defense systems.

## Metabolic profiling focused on interactions between plant and microbe

Metabolic profiling is the characterization and quantification of low-molecular weight metabolites and their intermediates in biological systems (Roessner and Bowne, 2009). This profiling aims to capture metabolites involved in the dynamic plant response to genetic modification, growth and developmental manipulation, and biotic/abiotic stresses (Clarke and Haselden, 2008). During plant-pathogen interaction, pathogens attempt to utilize the metabolism of host plants to suppress plant defense and to obtain nutrients (Dangl and Jones, 2001; Chisholm et al., 2006; Collinge, 2009). Metabolites that are synthesized by a host plant during a plant-microbe interaction can serve as signals, sedatives, or toxins to either aid the association with the microbe, or to attempt to limit the proliferation of the microbe (Thomma et al., 2002; Krishnan et al., 2005; Allwood et al., 2010, 2011; Schwessinger et al., 2012). Ultimately, most metabolic profiling will aim to capture *in situ* changes in cellular output in a spatially or temporal discrete region (Sumner, 2006; Timischl et al., 2008; Sumner et al., 2011; Khakimov et al., 2012). In the case of plant-pathogen interactions, profiling generally focuses on the plants metabolic response. Assaying microbial metabolites that are involved in plant-microbe interaction remains challenging. Assigning signals produced by a microbe requires separating them from those of the host plant and when grown in isolation, their metabolic output may not reflect a pathogenic state. When considering the plant cell wall, the relative predictability of metabolites in specific tissues provides an excellent starting point for looking at metabolic shifts associated with microbial ingress.

### Analysis of plant cell wall polysaccharides through metabolic profiling

At a broad scale, measurement of cell wall metabolites has been well-defined for decades. Neutral and acidic polysaccharides (Blakeney et al., 1983), acid insoluble and soluble glucose (Updegraff, 1969), soluble and insoluble lignin fractions (NREL, 2000) and the linkages between glycosyl units (Tong and Gross, 1988) can be examined with spectrophotometric, high performance liquid chromatography (HPLC), or gas chromatography (GC) coupled to mass spectroscopy (GC MS) to obtain a snapshot of the cell wall composition (Kopka, 2006). Similarly, at a much higher resolution, the structure of cell wall polysaccharides can be examined by uniformly feeding the plant with a isotope trace (^13^C-glucose and ^15^N-ammonia) and then employing 13C-magic angle spinning solid state nuclear magnetic resonance spectroscopy (SS-NMR) (Dick-Pérez et al., 2011; Fernandes et al., 2011; Harris et al., 2012). However, the capacity to look at spatially discrete regions of cell wall composition, which are linked to microbial association, can be difficult due to the relatively large amount of material needed for many of these techniques. To get around this limitation, combining systematic metabolite profiling with immunological approaches can be effective. For example, immunological approaches have been used to investigate the glycome profiling of wide array of plant cell wall polysaccharides (Pattathil et al., 2010, 2012; DeMartini et al., 2011; Fangel et al., 2012). Currently, around 150 antibodies that can recognize diverse epitopes present on each of the major classes of plant polysaccharides exist and are continuing to be developed. These antibodies have been used for *in situ* localization of epitopes to further our understanding of cell wall composition (Pattathil et al., 2012). Carbohydrate Microarray Polymer Profiling (CoMPP) has been streamlined as a screening platform to analyze cell wall polysaccharides by combining the specificity of monoclonal antibodies with a high-throughput microarray system (Alonso-Simón et al., 2009; Moller et al., 2012). In the context of microbial ingress, antibody based polysaccharide visualization has been utilized to observe altered xyloglucan arising from infection by the fungal pathogen *Botrytis cinerea* (Nguema-Ona et al., 2012, 2013). While difficulties arise in assigning quantitative data for localized metabolite profiles via immunological techniques, the capacity to gain unparalleled qualitative data is emerging. Additionally, a versatile high-resolution oligosaccharide microarray has been developed for cell wall analysis, which aids in the validation and characterization of target oligosaccharides produced via hydrolysis of polysaccharides or *de novo* synthesis (Pedersen et al., 2012). This library of cell wall oligosaccharides has been created by coupling target oligosaccharides with cognate proteins to form neoglycoconjugates, which in turn can be printed onto a microarray format (Pedersen et al., 2012). One can imagine the importance of such techniques to identify and characterize oligosaccharides identified during metabolic profiling.

Other techniques for assessing metabolites on a screening scale include Oligosaccharide Mass Profiling (OLIMP) coupled with Matrix-Assisted Laser Desorption/Ionization Time Of Flight (MALDI-TOF)-MS (Obel et al., 2009), or using of a suite of 74 polysaccharide degrading enzymes (Bauer et al., 2006). Both techniques were developed for the small-scale assessment of plant cell wall polysaccharides and to examine the oligosaccharides formed from polysaccharides that are digested by specific degrading enzymes (Bauer et al., 2006; Obel et al., 2009). OLIMP has particularly high sensitivity, thus making it ideal for small samples. It needs short preparation time and is suitable for *in situ* wall analysis at the cellular level. Importantly, OLIMP enables the comparative analysis of the wall polymers in a Golgi-enriched fraction vs. the apoplast fraction based on matrix polysaccharides, which may extend information about cellular functions during plant-pathogen interaction. OLIMP has been used to examine microbial alterations of the cell wall (Lionetti et al., 2007; Manabe et al., 2011), and allowed researchers to pinpoint that the alteration in esterification of pectin and xylan influenced the outcome of *B. cinerea* infection.

## Cell imaging and spetroscopic techniques

Advanced cellular imaging can be useful to investigate phenotypes linked to plant-microbe associations. Cellular imaging can be particularly important when applying a quantitative methodology to imaging techniques. Many microscopic techniques are available, including light (Wilt et al., 2009), fluorescence (Lichtman and Conchello, 2005), and confocal microscopy (Nwaneshiudu et al., 2012). However, the outcomes of cell imaging can be influenced by many factors, such as microscope resolution, the rate at which images can be acquired, cell type being examined and the abundance/size of the tagged protein or structure being observed (Stephens and Allan, 2003; Shaw, 2006; Table 1 for more details). Live cell imaging techniques (Table 1) have facilitated our understanding of plant cell wall dynamics in several different applications (Lee et al., 2011; Sappl and Heisler, 2013), and have been broadly applied when studying specific aspects of cell wall alteration during the interaction between a host plant and microbe.

**Table 1:** **Comparison of different cell imaging/spectroscopy methods for plant cell wall study**.

**Technique**	**Acquisition speed**	**Cell damage**	**Labeling (Fluorescence/ Coating/Staining)**	**Live cells**	**Single cell detection**	**Spatial/ Temporal resolution**	**Sample preparation**	**Chemical composition analysis**	**Measured parameter/information provided/limitation or possible problem caused**	**References**
Bright-field microscopy (BFM)	Slow	Yes	No	Yes	Yes	Low ~2–3 μm	Often complex	Not available	Particle shape and size, cell-wall surfaces, and multilamellar architecture	Lacayo et al., 2010; Moran-Mirabal, 2013
Fluorescence microscopy (FM)	Fast	No	Yes	Yes	Yes	High ~10–255 nm	Easier	Not available	3D-cell wall structure, relative amount of cell wall polymers among different cells, localization and interactions of different wall components; photobleaching	Shaw, 2006; Frigault et al., 2009; Lacayo et al., 2010
Confocal laser scanning microscopy (CLSM)	Slow	No	Yes	Yes	Yes	High ~0.2–0.8 μm	Easier	Not available	3D-cell wall structure, localization and interactions of different wall components, multiple labels usage, focus to small regions; Scanning speed limits	McCann et al., 2001; Stephens and Allan, 2003; Ma et al., 2013
Spinning disk confocal microscopy (SDCM)	Very fast for single color acquisition	No	Yes	Yes	Yes	High ~0.2–0.8 μm	Easier	Not available	3D-cell wall structure, broad laser focus, quantitative analysis of polymer dynamics; switching between laser lines limit the acquisition speed	Stephens and Allan, 2003; Paredez et al., 2006; Bischoff et al., 2009
Transmission electron microscopy (TEM)	Fast	Yes	Yes	No	Yes	High ~0.2–10 nm	Time and skill demanding	Not available	Cell-wall surfaces and multilamellar architecture, cell wall ultrastructural organization; Small sample areas, high resolution	Kristensen et al., 2008; Sant′Anna et al., 2013
Scanning electron microscopy (SEM)	Fast	Yes	Yes	No	Yes	High ~1–4 nm	Time and skill demanding	Not available	Cell-wall surfaces and multilamellar architecture, uses atom-coated surfaces to determine topologies	Sarkar et al., 2009; Donohoe et al., 2011
Localization microscopy (LM)	Fast	Yes	Yes	Yes	Yes	High ~2–25 nm	Easier	Not available	3D-cell wall structure, single-molecule localization, super resolution techniques, nanoscale glucan polymer analysis	Betzig et al., 2006; Eggert et al., 2014
Fourier transform infra-red (FTIR) microspectroscopy/Raman microspectroscopy	Slow	No	No	Yes	Yes	High ~250 nm	Time and skill demanding	Available	Multiple components chemical analysis, and orientation of the cellulose microfibrils. The results are significantly influenced by the environment and water. Spectra are difficult to analyze and interpret	Chen et al., 1997b; Agarwal et al., 2010; Gierlinger et al., 2012
Atomic force microscopy (AFM)	Fast	No	No	Yes	Yes	High ~0.1–30 nm	Easier	Available	3D-cell wall structure, topology of the cell wall surface; poor chemical resolution	Kirby et al., 1996; Zhang et al., 2012
X-RAY diffraction /neutron diffractometry	Fast	No	No	Yes	Yes	High ~0.05–0.4 nm	Time and skill demanding	Available	3D cell wall structure, degree of crystallinity, crystal size, chain orientation; only use for oriented crystal polymers	Burgert, 2006; Lacayo et al., 2010; Park et al., 2014
Nuclear magnetic resonance (NMR) spectroscopy	Fast	No	No	Yes	Yes	High ~10–90 nm	Time demanding	Available	Molecular dynamics, crystal structure, cellulose orientation; Interference could be caused by high molecular weight polymers, range of temperatures and frequencies are limited	Chylla et al., 2013; Wang et al., 2013

### Analysis of cell wall structure and function with cell imaging techniques

There are several techniques that may be used to investigate structural and functional changes of plant cell walls during plant-microbe interactions. Aside from examining the phenotype, actually pinpointing defects in the cell wall often requires the merger to two or more techniques, including profiling cell wall structure as described above. Electron microscopy, both scanning (SEM) and transmission (TEM), along with fluorescence microscopy (FM) in the form of laser scanning or spinning disk confocal microscopy are of particularly interest. These techniques have been used together to examine plant-microbe interaction through alterations in the cell wall. Here, FM and TEM (Table 1) revealed that multi-vesicular bodies participated in cell wall-associated defense to powdery mildew in barley (An et al., 2006). As individual techniques, neither could ascertain mechanistic association, but together these techniques allowed a snapshot of inter and intracellular occurrences. Further, the relevance of plasma membrane–cell wall adhesion for cowpea resistance to rust fungi penetration was pinpointed by an integrated use of light and confocal microscopy (Mellersh and Heath, 2001). Laser scanning confocal microscopy can be used to track both plant and microbial proteins in live tissue. For example, confocal microscopy was used to track the dynamics of a *Xanthomonas* outer protein J (XopJ) in tobacco plants (Table 1), and revealed its interference (alteration of intracellular vesicle trafficking and polarized protein secretion) with cell wall-associated defense responses (Bartetzko et al., 2009). Similarly, but for a plant protein, the application of spinning disk confocal microscopy allowed the visualization of the CELLULOSE SYNTHASE (CeSA) complexes after exposure to the dinitrite-peptide Thaxtomin-A, which is a phytotoxin produced by *Streptomyces scabies* and *S. eubacteria* (Bischoff et al., 2009). Confocal microscopy allows the user to observe the microbial effector while it influences the target plant protein or cellular process. We recently utilized a screen of microbial endophytes (Xia et al., 2013) to identify microbial factors that induce cellulose inhibition and identified the compound acetobixan from a *Bacillus* sp. (Xia et al., 2014). Confocal microscopy allowed us to validate that the target process that the microbe was altering in the host plant was cellulose biosynthesis, which revealed a specific mechanism for this association.

The mechanisms of plant cell wall organization and dynamics have been extensively studied, and the use of suitable chemical probes to examine cell wall polysaccharide organization is expanding (Vorwerk et al., 2004; Lee et al., 2011). Recently, small molecule probes that bind to polysaccharides with high resolution and sensitivity have been developed (Knox, 2008; Pattathil et al., 2010; Lee et al., 2011), particularly in the form of click chemistry (Wallace and Anderson, 2012). An example of this approach was the utilization of an alkynylated fucose analog (FucAl) incorporated into the cell wall pectin fraction to elucidate pectin delivery, architecture, and dynamics in Arabidopsis (Anderson et al., 2012). Ultimately, the development of small molecule probes compatible with live-cell imaging can further enhance the understanding of fundamental biological questions pertinent to the cell wall during plant-microbe interaction, and can even be targeted to specific events.

### Additional imaging techniques of note

Atomic force microscopy (AFM) is a technique with expanding use and potential. The extremely high resolution of AFM can allow the examination of events occurring within the nm scale. AFM has recently been used to detect the interaction of a synthetic carbohydrate-binding module with plant cellulose, and the structural changes of crystalline cellulose at a cell-wall surface (Zhang et al., 2012, 2013). In terms of plant microbe interactions, AFM recently provided nanoscale imaging of cell surfaces in their native state and revealed cell wall dynamics and modification during Arabidopsis and *Fusarium oxysporum* interaction (Adams et al., 2012). These selected studies underline the necessity to utilize the ever-expanding technological advances in imaging systems, often in concert with metabolic profiling, to maximize the detail of the investigation.

Localization microscopy, which is a form of super-resolution microscopy, focuses on the localization of single fluorescent molecule. Such super-resolution microscopy has been used to analyze the infection site of the fungal pathogen powdery mildew on Arabidopsis plants at a nanoscale level (Eggert et al., 2014). The technique was sensitive enough to show that the microbial pathogen induced the synthesis of the (1,3)-β-glucan cell wall polymer callose, which interacted with the (1,4)-β-glucan cellulose to form a three-dimensional network for preventing pathogen infection. The formation of callose associated with pathogen ingress has been well-studied, but such inter-polymer associations could not have been proven without technical breakthroughs. It will be interesting to see whether such techniques are combined with click-chemistry to observe an increasing number of interactions simultaneously.

The combined approaches of microscopy with spectroscopy can also facilitate the investigation of wall associated ultra-structure modifications, and the chemical compositions of the plant cell wall during plant-pathogen interaction (Table 1 for more details). Fourier Transform Infrared (FT-IR) spectromicroscopy has been used to determine the presence and orientation of functional groups of cellulose and pectin in plant cell walls for well over a decade (Chen et al., 1997a,b; Kaèuráková et al., 2000; Wilson et al., 2000). This technique was used to show that a mutation in Arabidopsis *PMR6*, which encodes a pectate lyase-like protein and is required for the growth and reproduction of plant fungal powdery mildew pathogen-*Erysiphe cichoracearum*, altered plant cell wall composition by increasing pectin accumulation. Both absorbance peaks attributed to cellulose and xyloglucan shifted down in energy and broadened in the spectra of *pmr6-1* cell walls, which indicated that either the –CH2OH group or the hydrogen bond of cellulose in *pmr6-1* had been changed (Vogel et al., 2002).

Raman microscopy (Inelastic scattering with a photon from a laser light source) combined with FT-IR spectromicroscopy (Photon absorption) can facilitate the observation of ultra-structure, such as cellulosic crystals on the micro-scale (<0.5 μm) level (Agarwal et al., 2010), as well as the alignment and orientation of cellulose microfibrils with respect to the fiber axis between different cell wall layers (Gierlinger et al., 2012). This combined approach also improves our ability to visualize and analyze the chemical composition of plant cell walls. For instance, the spectra of the two wall-matrix polymers: lignin and pectin display discernable marker bands, which do not overlap with the cellulose signature, so their distribution in the plant cell wall can be easily visualized, imaged, and analyzed using these techniques (Gierlinger and Schwanninger, 2006; Richter et al., 2011; Gierlinger et al., 2012).

## Prospects

Recent technical breakthroughs in combining higher resolution imaging and metabolic profiling techniques have yielded numerous discoveries in how plant cell wall function is modulated during microbial interaction. Although much effort was spent to be inclusive in this mini-review, due to space constraints we apologize for excluding numerous developing techniques not limited to but including those associated with biochemical pull downs, protein-protein interaction arrays and more. Although advanced cell imaging and spectroscopic techniques have facilitated such studies, the recent identification of the enormously complex phytobiome (Bulgarelli et al., 2012; Lundberg et al., 2012, 2013) reveals an outstanding question of the function of the phytobiome in plant-microbe associations. The use of next generation sequencing has revealed that many more microbes are present within plant tissue than those previously identified as obligate endophytes. It remains unclear how these microbial mutualists are associating with (or avoiding) the plant cell wall and associated defense pathways, and whether under pathogen interaction, they matter?

### Conflict of interest statement

The authors declare that the research was conducted in the absence of any commercial or financial relationships that could be construed as a potential conflict of interest.
